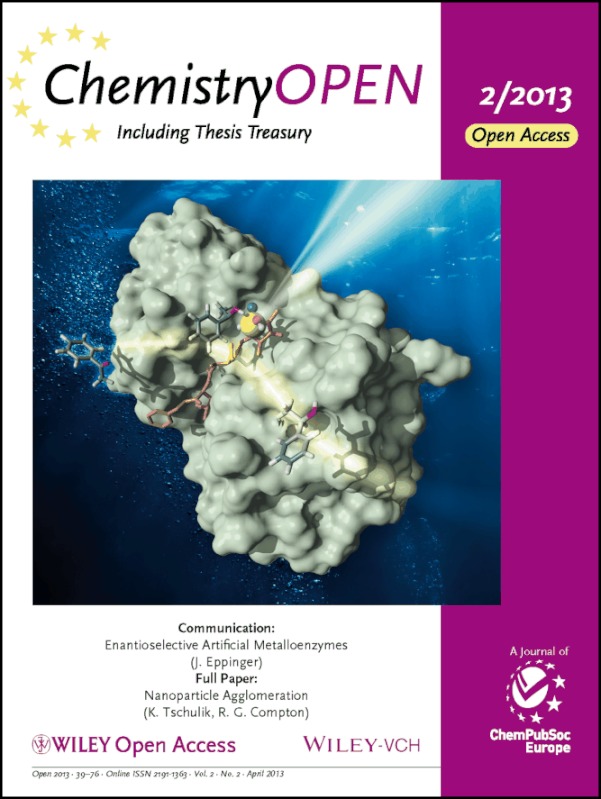# Cover Picture: Metal-Conjugated Affinity Labels: A New Concept to Create Enantioselective Artificial Metalloenzymes (ChemistryOpen 2/2013)

**DOI:** 10.1002/open.201390005

**Published:** 2013-04-23

**Keywords:** affinity labels, artificial metalloproteins, asymmetric catalysis, cysteine proteases, hydrogenation

## Abstract

**Cover Picture:**

Thomas Reiner, Dominik Jantke, Alexander N. Marziale, Andreas Raba, and Jörg Eppinger*

**The cover picture illustrates** the concept of using metal-conjugated affinity labels (m-ALs) to convert proteases into well-defined and catalytically active artificial metalloenzymes. The X-ray structure of the papain-bound inhibitor E64c (orange) served as the basis to predict the orientation of the half-sandwich rhodium(III) moiety (yellow sphere) within the binding pocket of the protease. The well-defined position of the affinity label on the protein surface leads to a distinct environment of the metal center, which translates into enantiomeric ratios of up to 82:18 in the aqueous hydrogenation of ketones. For more details, see the Communication by Jörg Eppinger et al., on p. 50 ff.